# Curative Therapies for Hemophilias and Hemoglobinopathies in Adults: Immune, Gene, and Stem Cell Approaches in a Global Context

**DOI:** 10.3390/biomedicines13082022

**Published:** 2025-08-19

**Authors:** Ayrton Bangolo, Behzad Amoozgar, Lili Zhang, Sarvarinder Gill, Daniel Lushimba Milolo, Justin Ngindu Kankonde, Claude Mbuyi Batakamuna, Robert Tassan, Christina Cho, John Bukasa-Kakamba, Kelley Mowatt-Pesce

**Affiliations:** 1 Department of Hematology and Oncology, John Theurer Cancer Center at Hackensack University Medical Center, Hackensack, NJ 07601, USA; behzad.amoozgar@hmhn.org (B.A.); lili.zhang@hmhn.org (L.Z.); sarvarinder.gill@hmhn.org (S.G.); 2 Department of Internal Medicine, Université Notre-Dame du Kasayi, Kananga P.O. Box 70, Democratic Republic of the Congo; danlushimba3@gmail.com (D.L.M.); justinngindu@gmail.com (J.N.K.); cmbuyi918@gmail.com (C.M.B.); 3 Division of Classical Hematology, John Theurer Cancer Center at Hackensack University Medical Center, Hackensack, NJ 07601, USA; robert.tassan@hmhn.org (R.T.); kelley.mowattpesce@hmhn.org (K.M.-P.); 4 Division of Stem Cell Transplant and Cellular Therapy, John Theurer Cancer Center at Hackensack University Medical Center, Hackensack, NJ 07601, USA; christina.cho@hmhn.org; 5 Department of Endocrinology and Metabolic Diseases, University of Kinshasa, Kinshasa 999069, Democratic Republic of the Congo; johnbukasa73@gmail.com; 6 Department of Endocrinology, Liege University Hospital Center, 4000 Liège, Belgium

**Keywords:** gene therapy, hemophilia, sickle cell disease, β-thalassemia, CRISPR, emicizumab, hematopoietic stem cell transplant, global health equity, viral vectors, genome editing

## Abstract

Hemophilias and hemoglobinopathies—including hemophilias A and B, sickle cell disease (SCD), and β-thalassemia—are debilitating genetic disorders associated with significant global health burdens. While traditional management has centered on factor replacement and transfusions, these approaches remain palliative, with limited access and durability in many regions. Recent advances in immune-based therapeutics (e.g., emicizumab, concizumab, crizanlizumab), viral vector-mediated gene addition (e.g., Roctavian, Hemgenix), and gene-modified autologous stem cell therapies (e.g., Zynteglo, Casgevy) have ushered in a new era of disease-modifying and potentially curative interventions. These therapies offer durable efficacy and improved quality of life, particularly in adult populations. However, implementation remains uneven across global health systems due to high costs, limited infrastructure, and regulatory heterogeneity. Additionally, ethical considerations such as long-term surveillance, informed consent in vulnerable populations, and social perceptions of genetic modification present ongoing challenges. Innovations such as multiplex genome editing, immune-evasive donor platforms, synthetic biology, and AI-driven treatment modeling are poised to expand therapeutic horizons. Equitable access, particularly in regions bearing the highest disease burden, will require collaborative funding strategies, regional capacity building, and inclusive regulatory frameworks. This review summarizes the current landscape of curative therapy, outlines implementation barriers, and calls for coordinated international action to ensure that transformative care reaches all affected individuals worldwide.

## 1. Introduction

Hemophilias and hemoglobinopathies are inherited hematologic disorders that exert a substantial burden on global health, particularly in low- and middle-income countries (LMICs) where access to diagnosis and treatment remains limited. Hemophilias A and B are X-linked bleeding disorders caused by deficiencies of factors VIII and IX, respectively, while hemoglobinopathies, including sickle cell disease (SCD) and β-thalassemia, arise from mutations in the genes encoding the β-globin chain of hemoglobin. Von Willebrand disease (vWD), the most common inherited bleeding disorder, further complicates the spectrum of congenital coagulopathies, with varying degrees of severity depending on its type. Worldwide, it is estimated that over 300,000 infants are born annually with severe hemoglobinopathies, and more than 400,000 individuals live with hemophilia, with the true numbers likely higher due to underdiagnosis and underreporting in resource-poor regions [1,2].

While advances in supportive and prophylactic care—such as factor replacement therapy, hydroxyurea, and blood transfusions—have improved survival and quality of life, these approaches remain largely palliative and are associated with significant limitations. Chronic transfusions carry risks of iron overload and alloimmunization; factor replacement is costly and may result in the development of inhibitory antibodies. For many patients, particularly adults with advanced disease complications or in countries with limited access to comprehensive care, these treatments fail to offer long-term disease control or meaningful quality-of-life restoration. Consequently, there is a growing demand for transformative, curative therapies that address the underlying pathophysiology of these disorders.

Curative approaches for hemophilias and hemoglobinopathies have emerged through decades of research in hematopoietic stem cell transplantation (HSCT), gene therapy, and, more recently, immune-based modulation. HSCT has demonstrated curative potential in both sickle cell disease and β-thalassemia, particularly when performed with HLA-matched sibling donors, although challenges such as graft-versus-host disease (GVHD) and transplant-related mortality persist. Advances in reduced-intensity conditioning regimens and haploidentical transplantation have extended its feasibility to older patients and those without matched donors [3]. Gene therapy, particularly lentiviral vector-based and CRISPR/Cas9-mediated genome editing strategies, has opened new frontiers in reactivating fetal hemoglobin or correcting genetic defects at their source [4,5].

In hemophilia, gene therapy has progressed from preclinical experiments to pivotal clinical trials, with AAV-mediated gene transfer of factors VIII and IX showing sustained expression and reduction or elimination of bleeding episodes in adult patients. For instance, valoctocogene roxaparvovec (an AAV5-hFVIII-SQ therapy) and etranacogene dezaparvovec (AAV5-Padua hFIX) have demonstrated clinical efficacy, leading to conditional approvals in Europe and regulatory evaluation in other regions [6,7]. These interventions represent a paradigm shift in the management of hemophilia, offering the potential for a one-time treatment with durable benefits, though long-term data and accessibility in non-high-income settings remain evolving challenges.

Immune modulation strategies have also gained interest, especially for managing inhibitors in hemophilia or enhancing engraftment in transplantation. Agents such as emicizumab—a bispecific monoclonal antibody mimicking factor VIII—have proven effective even in patients with inhibitors and have been transformative in prophylaxis [8]. In hemoglobinopathies, emerging immunotherapies aim to ameliorate inflammatory complications and vaso-occlusive crises by targeting cellular adhesion molecules and cytokine pathways, although these remain largely adjunctive rather than curative.

Despite these therapeutic advances, significant disparities persist in access to curative care across the globe. Most gene therapy trials and transplant centers are concentrated in high-income countries, while patients in sub-Saharan Africa, South Asia, and parts of Latin America continue to face early mortality and poor quality of life due to resource limitations and delayed diagnosis [9,10]. Therefore, integrating curative strategies into a global context necessitates attention not only to biomedical innovation but also to policy, equity, and infrastructure development, ensuring that transformative therapies reach all who need them, regardless of geography or socioeconomic status.

## 2. Disease Background

### 2.1. Hemophilias A and B

Hemophilias A and B are rare, X-linked recessive bleeding disorders caused by mutations in the F8 and F9 genes, respectively, resulting in deficiencies of factor VIII or IX. These coagulation factors play critical roles in the intrinsic pathway of hemostasis. In their absence, thrombin generation is impaired, leading to prolonged bleeding episodes, particularly into joints and muscles. The clinical severity correlates with factor activity: severe (<1%), moderate (1–5%), and mild (>5%) forms exhibit increasing spontaneous or trauma-induced bleeding tendencies. Genetic mutations include inversions, missense/nonsense mutations, and large deletions or duplications [11].

Historically, hemophilia was managed using whole blood or fresh frozen plasma, evolving to cryoprecipitate and plasma-derived factor concentrates. By the 1990s, recombinant factors VIII and IX products revolutionized treatment, though their frequent administration posed challenges due to short half-lives. This led to the development of extended half-life (EHL) products, which incorporate modifications like PEGylation or Fc-fusion to prolong circulation time. These EHL therapies reduce infusion frequency and improve prophylaxis adherence, significantly decreasing annualized bleeding rates in adults with hemophilia [11].

Current treatment includes on-demand therapy for acute bleeds and prophylactic regimens aimed at maintaining factor levels above a threshold to prevent spontaneous bleeding. However, inhibitor development, particularly in severe hemophilia A (~30% of patients), remains a major complication. Inhibitors are alloantibodies that neutralize infused factor VIII or IX, rendering standard replacement therapies ineffective. Management strategies in such cases include immune tolerance induction (ITI), recombinant factor VIIa, and emicizumab—a bispecific monoclonal antibody that mimics the function of factor VIII. Emicizumab has transformed care for patients with and without inhibitors by offering subcutaneous administration and sustained hemostatic control [12,13].

Despite these advances, challenges persist in global access, affordability, and lifelong treatment adherence. Moreover, long-term safety data for EHL and non-factor products remain under evaluation. Real-world studies and registry data are increasingly important in characterizing their efficacy across diverse adult populations and informing strategies for prophylaxis initiation, transition from standard products, and individualized dosing [14]. Novel agents in the pipeline include siRNA therapies targeting antithrombin (e.g., fitusiran), anti-TFPI antibodies (e.g., concizumab), and gene therapy, which may offer a path to functional cure [15,16].

### 2.2. Rare Congenital Coagulation Factor Deficiencies

Rare bleeding disorders (RBDs) encompass deficiencies of factors I, II, V, VII, X, XI, and XIII and combined deficiencies, each resulting from autosomal recessive inheritance and manifesting with variable bleeding phenotypes. While individually uncommon, collectively they affect thousands of patients worldwide. Diagnosis requires specialized coagulation assays, often unavailable in resource-limited settings, leading to under-recognition and suboptimal management. Recent population-based studies have emphasized the need for dedicated registries and global collaborative efforts to better understand and treat these disorders [17].

Factor XI deficiency (hemophilia C), more common in Ashkenazi Jewish populations, typically presents with mild to moderate bleeding, especially after trauma or surgery. There is no standardized prophylaxis regimen, and treatment decisions are often individualized. Options include antifibrinolytics, FFP, or factor XI concentrate where available. Emerging studies have explored FXI antisense oligonucleotides (e.g., IONIS-FXI-LRx) not only for bleeding risk reduction but also for thromboembolism prevention in high-risk settings [18].

Factor VII deficiency, the most common RBD, is associated with mucocutaneous and postoperative bleeding. Recombinant activated factor VII (rFVIIa) remains the standard of care, although dosing strategies are not well standardized. Similarly, factor X deficiency, associated with spontaneous CNS and GI hemorrhages, is treated with plasma-derived FX concentrates or PCCs. Advances in recombinant formulations for both FVII and FX are under investigation, with recent clinical data demonstrating favorable pharmacokinetics and reduced immunogenicity [19].

Factor XIII deficiency, though exceedingly rare, is associated with umbilical cord bleeding, intracranial hemorrhage, and impaired wound healing. Lifelong prophylaxis with FXIII concentrate has been shown to prevent severe bleeding. Other rare factor deficiencies (V, II, fibrinogen) are treated using FFP, cryoprecipitate, or specialized concentrates. Recent studies underscore the importance of genotype–phenotype correlations, personalized replacement thresholds, and long-term registry participation to advance evidence-based treatment guidelines for RBDs [20].

### 2.3. Von Willebrand Disease

First described in 1926 by Erik von Willebrand, von Willebrand disease (vWD), the most prevalent inherited bleeding disorder, results from defects in von Willebrand factor (vWF), a multimeric glycoprotein involved in platelet adhesion and FVIII stabilization. vWD is classified into three main types: type 1 (partial quantitative deficiency), type 2 (qualitative dysfunction), and type 3 (complete deficiency). Type 2 is further divided into subtypes (2A, 2B, 2M, and 2N) based on functional characteristics. Recent genomic studies have improved diagnostic accuracy by identifying pathogenic variants in the VWF gene and characterizing multimeric abnormalities [21].

Diagnosis of vWD involves vWF antigen and activity assays (ristocetin cofactor or GPIb binding), FVIII levels, and bleeding scores. Advances in laboratory diagnostics, such as VWF:GPIbM assays and vWF multimer analysis, allow better subtype differentiation. Genetic testing has become increasingly relevant, especially in types 2N and 3 vWD. Nevertheless, variability in assay availability and interpretation continues to pose diagnostic challenges in many settings [22].

Acute bleeding is managed with desmopressin (DDAVP) in responsive patients (mainly type 1 and some 2A), and with plasma-derived vWF/FVIII concentrates in unresponsive or severely affected individuals. For perioperative prophylaxis and pregnancy, tailored dosing is critical to minimize bleeding and thrombotic complications. Recent clinical trials have validated population pharmacokinetics models to guide individualized dosing, improving outcomes in adult women with vWD during labor and delivery [22].

Acquired vWD (AVWD), a non-inherited disorder, arises secondary to lymphoproliferative diseases, cardiovascular disorders, or autoantibodies. Management includes treatment of the underlying condition, immunosuppression, IVIG, or vWF concentrate, depending on etiology. Recent case series suggest that newer agents like emicizumab may play a future role in select AVWD patients, although controlled data are lacking [23]. Given its phenotypic overlap with other coagulopathies, AVWD requires high clinical suspicion and multidisciplinary coordination for effective diagnosis and treatment.

### 2.4. Hemoglobinopathies

Hemoglobinopathies are divided into quantitative (thalassemias) and qualitative (e.g., sickle cell disease) disorders affecting hemoglobin synthesis or structure. β-thalassemia arises from mutations in the HBB gene, leading to absent or reduced β-globin production. The imbalance between α- and β-globin chains causes ineffective erythropoiesis, hemolysis, and chronic anemia. In contrast, sickle cell disease (SCD) results from a missense mutation (Glu6Val) in β-globin, promoting polymerization of deoxygenated hemoglobin S and causing vaso-occlusion, hemolytic anemia, and multiorgan damage [24].

The standard of care for SCD includes hydroxyurea, which increases fetal hemoglobin (HbF), thereby reducing polymerization of HbS. More recent agents include L-glutamine, which reduces oxidative stress and vaso-occlusive crises; crizanlizumab, a monoclonal antibody targeting P-selectin to reduce cellular adhesion; and voxelotor, which stabilizes oxygenated hemoglobin to prevent polymerization. These agents have expanded the therapeutic landscape, though access, cost, and long-term outcome data remain barriers to widespread adoption [24,25].

Transfusion therapy is critical for managing acute complications (e.g., stroke, ACS) and in β-thalassemia major, where lifelong transfusions are often required. However, repeated transfusions result in iron overload, necessitating chelation therapy. Deferasirox is the most widely used oral chelator, though adherence remains a concern. Iron-related organ damage remains a major cause of morbidity and mortality, particularly in adults with β-thalassemia or transfusion-dependent SCD [26].

Exchange transfusions are often preferred in acute stroke, acute chest syndrome, and preoperative management in SCD. They reduce HbS levels more effectively while minimizing iron burden. Advances in automated erythrocytapheresis and vascular access techniques have made this approach more feasible, even in outpatient settings. Nonetheless, logistical challenges and complications such as alloimmunization, delayed hemolytic reactions, and vascular access issues require careful long-term planning in adult patients [27].

## 3. Immune-Based Therapeutics

### 3.1. Immune Tolerance Induction in Hemophilia (ITI)

Inhibitor development remains the most serious complication in hemophilia care, affecting nearly 30% of patients with severe hemophilia A and 3–5% with hemophilia B. Immune tolerance induction (ITI) remains the only proven strategy to eradicate inhibitors. The approach involves regular administration of high-dose factor VIII or IX over months to years to desensitize the immune system. Although traditionally used in pediatric populations, recent data support ITI in adults with newly developed inhibitors or relapse after prior success [28].

Recent studies have explored biomarkers predictive of ITI success, including inhibitor titers, peak historical titers, and genetic background (e.g., F8 mutation type). A prospective analysis by the GTH study group found that early ITI initiation and low peak inhibitor titers were associated with higher success rates. Furthermore, advances in pharmacokinetics-guided regimens and recombinant products with improved immunogenic profiles have contributed to better outcomes and reduced ITI duration [29].

Immune modulation strategies adjunctive to ITI, such as the use of immunosuppressants or rituximab, have shown potential in patients with poor prognostic indicators or previous ITI failure. A review emphasized the utility of combining ITI with immunomodulatory agents in selected high-risk patients, particularly those with anamnestic responses or high-titer persistent inhibitors [30].

For hemophilia B, ITI is often complicated by severe allergic reactions or nephrotic syndrome, making immune tolerance more challenging. Nevertheless, individualized protocols incorporating immunosuppressants and careful monitoring have shown promise in small cohorts. The development of non-factor therapies is expected to reduce the reliance on ITI, but it remains the gold standard for patients pursuing curative elimination of inhibitors [31].

There is also growing interest in ITI as a platform to improve the safety of emerging gene therapies. Patients with pre-existing inhibitors are often excluded from gene therapy trials, so establishing immune tolerance beforehand may enable broader eligibility. Preclinical studies have proposed ITI prior to AAV vector administration as a potential strategy to overcome neutralizing antibodies and T-cell responses [32].

Overall, ITI remains a cornerstone of immune-based care in hemophilia with inhibitors, with evolving techniques, predictive modeling, and adjunctive therapies helping to improve its success in adults and high-risk groups.

### 3.2. Monoclonal Antibodies and Bispecific Agents in Hemophilia

The development of monoclonal antibodies and bispecific constructs has transformed the treatment paradigm for patients with hemophilia A, especially those with inhibitors. These biologics function by bypassing defective components of the coagulation cascade or modulating immune responses, offering alternative strategies to conventional factor replacement. Their advantages include subcutaneous delivery, extended dosing intervals, and efficacy even in the presence of inhibitors [33].

Emicizumab, the first bispecific antibody approved for hemophilia A, bridges activated factor IX and factor X, mimicking the function of factor VIII. It has shown remarkable efficacy in reducing bleeding events in both inhibitor and non-inhibitor populations, and it remains effective regardless of endogenous FVIII inhibitor levels [12]. Several new bispecific and monoclonal agents are currently in development.

Agents such as mim8 (Novo Nordisk) aim to improve on emicizumab’s efficacy and pharmacokinetics. Preclinical and early clinical data suggest enhanced potency and longer duration of action with mim8, potentially enabling monthly or even less frequent dosing. Clinical trials are ongoing in both prophylactic and surgical settings [34].

Monoclonal antibodies targeting regulatory proteins such as tissue factor pathway inhibitor (TFPI) or antithrombin offer another bypass strategy. Concizumab, an anti-TFPI antibody, enhances thrombin generation by blocking TFPI’s inhibitory effect on factor Xa. Phase 3 studies (explorer7, explorer8) showed promising results in reducing annualized bleeding rates in patients with hemophilias A and B, with or without inhibitors [35,36].

The development of these agents raises new questions regarding optimal use in surgical settings, combination with other therapies, and monitoring of thrombotic risk. As more biologics enter clinical practice, head-to-head comparisons and pharmacoeconomic studies will be essential to guide clinician decision making [37].

Ultimately, monoclonal and bispecific agents represent a maturing class of therapeutics in hemophilia, offering personalized, immune-based strategies with increasing global adoption.

### 3.3. Emicizumab for Hemophilia A: Mechanism and Indications

**Figure 1:**
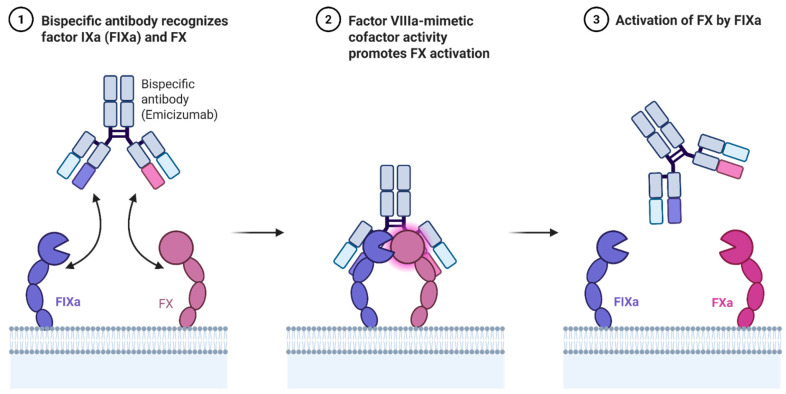
Mechanism of action of Emicizumab.

Emicizumab is a humanized bispecific IgG4 monoclonal antibody that binds activated factor IX (FIXa) and factor X (FX), thereby mimicking the cofactor function of factor VIII (Figure 1). Unlike FVIII, emicizumab is not inhibited by anti-FVIII antibodies, making it especially valuable for patients with inhibitors. Its subcutaneous administration and favorable pharmacokinetics have redefined prophylaxis for hemophilia A [34].

Indicated for routine prophylaxis in patients with hemophilia A with or without inhibitors, emicizumab is approved across all age groups. The HAVEN clinical trial program demonstrated significant reductions in bleeding rates, improvement in joint health, and quality of life. HAVEN 1–4 trials confirmed efficacy in both inhibitor-positive and inhibitor-negative populations, and recent long-term follow-up confirms sustained benefit with low immunogenicity [12].

Real-world evidence supports the durability and safety of emicizumab, even in adults with established joint damage or limited venous access. A 2025 multicenter registry analysis highlighted reductions in hospitalizations and emergency department visits following transition to emicizumab [38]. Patients with severe hemophilia A on emicizumab have experienced fewer spontaneous bleeds and improved treatment adherence.

Challenges remain, particularly in the perioperative setting. Because emicizumab does not fully replicate the temporal and spatial kinetics of FVIII, surgical hemostasis requires tailored use of bypassing agents such as rFVIIa, with careful thrombotic risk management. Recent guidelines recommend perioperative coordination between hematology and surgical teams to minimize complications [39].

Additionally, rare reports of thrombotic microangiopathy (TMA) and thrombotic events in patients receiving emicizumab with activated PCCs have prompted the development of clear dosing protocols and patient education strategies. Ongoing postmarketing surveillance aims to clarify the incidence and predictors of these complications [40].

Overall, emicizumab represents a transformative therapy for hemophilia A and continues to shape future therapeutic design through its innovative mechanism and strong clinical impact.

### 3.4. Anti-TFPI and Anti-ATIII Approaches: Fitusiran and Concizumab

Fitusiran is a small interfering RNA (siRNA) therapy targeting antithrombin (AT) mRNA in hepatocytes, reducing AT production and thereby rebalancing thrombin generation. This approach seeks to offset the deficiency in procoagulant activity by downregulating natural anticoagulants. Fitusiran is administered subcutaneously once monthly, and trials have demonstrated meaningful reductions in annualized bleeding rates in both hemophilias A and B with and without inhibitors [41].

Concizumab, on the other hand, is a monoclonal antibody against TFPI, which regulates the extrinsic pathway of coagulation by inhibiting FXa and the TF-FVIIa complex. By neutralizing TFPI, concizumab promotes thrombin generation through the extrinsic pathway. Phases 2 and 3 trials (explorer series) showed significant reductions in bleeding episodes, particularly in inhibitor-positive patients [42,43].

Both therapies offer prophylaxis via non-factor pathways, allowing broader application to patients with inhibitors and those ineligible for traditional ITI or factor replacement. However, safety signals have emerged. Clinical holds were temporarily placed on fitusiran and concizumab trials due to thrombotic events, prompting dose adjustments and revised monitoring protocols. Phase 3 trials have since resumed with favorable safety profiles under the updated regimens [43].

Importantly, these agents represent a paradigm shift from factor replacement to immune modulation and rebalancing therapies. This is particularly relevant for adult patients with long-standing disease, joint disease, or comorbidities where intravenous access is difficult or factor infusions are poorly tolerated. Ongoing trials are exploring their use in perioperative care and in combination with emerging gene therapies.

The potential for fixed-dose, infrequent administration and independence from FVIII/IX genotypes offers a new therapeutic horizon. Nevertheless, as these therapies progress toward licensure, cost, global accessibility, and long-term outcomes remain important research and policy concerns [44].

These agents highlight the growing role of immune manipulation and RNA-targeting therapeutics in hemophilia, extending the concept of immune-based therapy beyond inhibitor eradication into primary disease control.

### 3.5. Immune Modulation in Sickle Cell Disease (SCD) and Thalassemia

Immune system dysregulation plays a central role in the pathophysiology and complications of both SCD and thalassemia. In SCD, chronic hemolysis, ischemia–reperfusion injury, and oxidative stress lead to systemic inflammation, characterized by elevated cytokines (e.g., IL-6, TNF-α), neutrophil activation, and endothelial dysfunction. Thalassemia, particularly in transfusion-dependent patients, also triggers immune activation via alloimmunization, chronic antigenic stimulation, and iron-induced oxidative stress [45].

Immunomodulatory therapies have emerged as promising adjuncts in managing these disorders, particularly in mitigating inflammatory complications and vaso-occlusive events (VOCs). For instance, corticosteroids have long been used acutely in SCD-related acute chest syndrome, though their use is limited by rebound effects and side effects. More recently, attention has turned to biologic agents targeting specific immune and inflammatory mediators involved in disease exacerbation [46].

Crizanlizumab, a monoclonal antibody against P-selectin, represents a key milestone in immune-based therapy in SCD. Approved in 2019, its efficacy in reducing the frequency of VOCs has been confirmed in adult trials, and further real-world data over the past five years have validated its use across a broader population, including those previously refractory to hydroxyurea [47,48].

Beyond P-selectin inhibition, research has investigated other immune targets, such as IL-1, IL-6, and complement pathways. Anakinra (IL-1 receptor antagonist) and tocilizumab (IL-6 receptor inhibitor) have been evaluated in early-phase trials for VOCs and systemic inflammation control, with preliminary evidence suggesting reduced pain and cytokine release [49]. Complement inhibitors, including eculizumab and newer C5a-targeting agents, have shown theoretical benefit in reducing hemolysis and endothelial activation, though clinical utility remains under investigation.

In β-thalassemia, immune modulation is particularly relevant in addressing alloimmunization, which complicates chronic transfusion regimens and limits compatibility. Recent studies advocate for early extended phenotypic matching and the use of immunosuppressive agents like rituximab in patients with multiple alloantibodies [50]. Furthermore, iron chelation appears to have immunomodulatory effects, with newer agents (e.g., deferiprone) showing favorable immune cell modulation profiles.

The integration of immune-based therapies in both SCD and thalassemia underscores a growing recognition of these disorders as inflammatory diseases with systemic immunologic consequences. Future therapies are likely to increasingly target immune pathways to prevent end-organ damage and reduce disease burden beyond cytotoxic or transfusion-based approaches.

### 3.6. Role of Inflammation in Vaso-Occlusion

Vaso-occlusive crises (VOCs), the hallmark of SCD, are now understood to be driven not only by mechanical obstruction from sickled erythrocytes but also by a complex inflammatory cascade involving leukocytes, platelets, endothelial cells, and cytokines. This paradigm shift has prompted the development of therapies targeting these inflammatory pathways [51].

In SCD, neutrophils are chronically activated and adhere to endothelium and erythrocytes, forming aggregates that obstruct blood flow. Endothelial activation, mediated by TNF-α, IL-1β, and reactive oxygen species, further amplifies leukocyte adhesion and thrombosis. Elevated levels of adhesion molecules such as P-selectin, E-selectin, and VCAM-1 are correlated with VOC frequency and severity [52].

The interaction of sickled RBCs with Toll-like receptors (TLRs) on innate immune cells has been shown to activate NF-κB signaling, triggering pro-inflammatory cytokine production. This chronic low-grade inflammation leads to endothelial dysfunction and contributes to complications such as pulmonary hypertension, nephropathy, and stroke [53].

Monocyte and macrophage polarization toward the M1 phenotype in SCD has also been linked to persistent inflammation. Recent studies have shown that hydroxyurea not only induces HbF but also modulates monocyte activation and reduces pro-inflammatory cytokine release, suggesting a dual mechanism of action [54].

In thalassemia, iron overload from transfusions exacerbates inflammation through the Fenton reaction and mitochondrial dysfunction, leading to increased IL-6 and CRP levels. Chronic activation of immune pathways contributes to ineffective erythropoiesis and bone marrow expansion, which are central to disease progression [55].

Thus, VOCs and long-term complications in hemoglobinopathies are deeply rooted in immunopathology. Ongoing research into the cellular and molecular drivers of inflammation offers promising therapeutic targets to prevent or attenuate these events.

### 3.7. Potential of Anti-Adhesion and Anti-Inflammatory Biologics

Biologics targeting adhesion molecules and inflammatory mediators have emerged as promising therapies for reducing vaso-occlusive events and chronic inflammation in SCD. The success of crizanlizumab (anti-P-selectin antibody) has spurred further investigation into similar strategies. By blocking P-selectin-mediated interactions between endothelial cells, platelets, and leukocytes, crizanlizumab reduces VOC frequency and hospitalization rates in both clinical trials and real-world settings [56].

Inclacumab, another anti-P-selectin monoclonal antibody, is currently under investigation in phase 3 trials. Preliminary results suggest it may offer similar or superior efficacy to crizanlizumab, with potentially fewer infusion-related reactions and longer dosing intervals [57]. Its role in secondary stroke prevention and post-acute pain crisis recovery is also being explored.

E-selectin and VCAM-1 inhibitors are in earlier stages of development. Small-molecule inhibitors and monoclonal antibodies targeting these molecules aim to disrupt leukocyte rolling and firm adhesion, thereby mitigating the early phases of VOC initiation. A novel anti-E-selectin agent (GMI-1271) has shown promise in preclinical studies and is being evaluated in hematologic malignancies and SCD [58].

**Figure 2:**
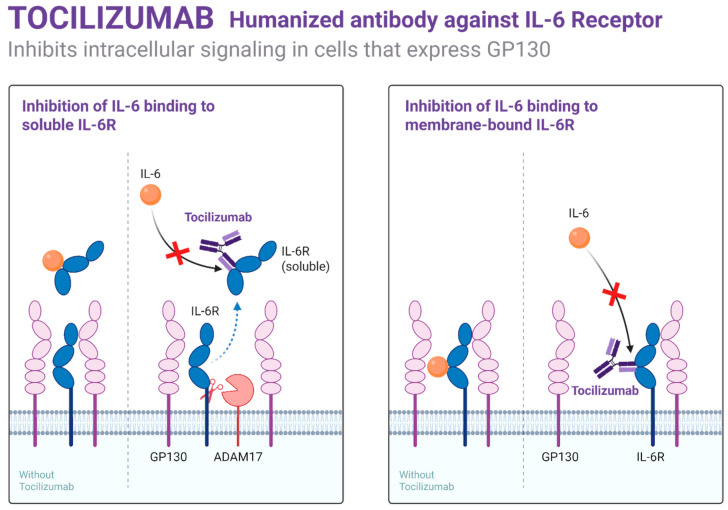
Mechanism of action of Tocilizumab.

Targeting cytokines such as IL-1β, IL-6, and TNF-α through biologics (e.g., canakinumab, tocilizumab), as shown in Figure 2, has yielded early clinical success in reducing VOCs and systemic inflammation. These agents are being studied both as monotherapies and adjuncts to hydroxyurea or gene-based therapies in adults with refractory disease [49].

Nanobody-based biologics and RNA-targeted therapies are also being explored for their ability to inhibit adhesion molecule expression or reduce endothelial activation. These approaches may offer oral administration or improved tissue penetration, expanding options for adult patients with poor venous access or compliance challenges [52].

As more adhesion-targeting agents become available, individualized therapy based on inflammatory biomarkers, VOC history, and comorbid inflammatory conditions may help personalize care and reduce long-term organ damage in hemoglobinopathy patients.

### 3.8. Immune Therapy in Acquired Von Willebrand Disease (AVWD)

Acquired von Willebrand disease (AVWD) is a rare, non-hereditary coagulopathy caused by autoimmune mechanisms, malignancy (particularly lymphoproliferative disorders), cardiovascular disease, or mechanical shear stress. In AVWD, autoantibodies, adsorption onto cell surfaces, or enhanced proteolysis leads to reduced plasma VWF levels and activity, mimicking inherited vWD [59].

The cornerstone of AVWD management is addressing the underlying cause. However, immunotherapy has gained prominence in cases of autoimmune or idiopathic AVWD. First-line therapies include corticosteroids, intravenous immunoglobulin (IVIG), and rituximab, especially in patients with autoantibody-mediated clearance of VWF. Recent reports show IVIG can transiently restore VWF levels in patients with lymphoproliferative disorders or MGUS-associated AVWD [60].

Rituximab, an anti-CD20 monoclonal antibody, has demonstrated success in inducing durable remission in AVWD associated with IgG-mediated clearance. Case series and small cohort studies over the past 5 years have supported its use in refractory AVWD and in patients unable to undergo splenectomy or chemotherapy [61].

Emerging immune therapies, such as BTK inhibitors and checkpoint inhibitors, may play a role in underlying lymphoid malignancies linked to AVWD. Their effect on AVWD resolution is currently under investigation, particularly in patients with Waldenström macroglobulinemia and AVWD overlap syndromes [62].

Plasma-derived VWF concentrates and recombinant therapies remain essential for managing acute bleeding or preparing for invasive procedures. However, immunomodulatory treatment is critical for achieving long-term hemostatic stability. Personalized regimens based on inhibitor titer, VWF recovery, and bleeding phenotype guide therapeutic choice [63].

Ongoing registry efforts and international collaborations are enhancing understanding of AVWD pathophysiology and optimizing immune therapy protocols. As diagnostic techniques improve, earlier identification of immune-mediated AVWD will enable more effective and targeted immune-based interventions.

## 4. Gene Therapy Approaches

### 4.1. Viral-Vector-Mediated Gene Addition

**Figure 3:**
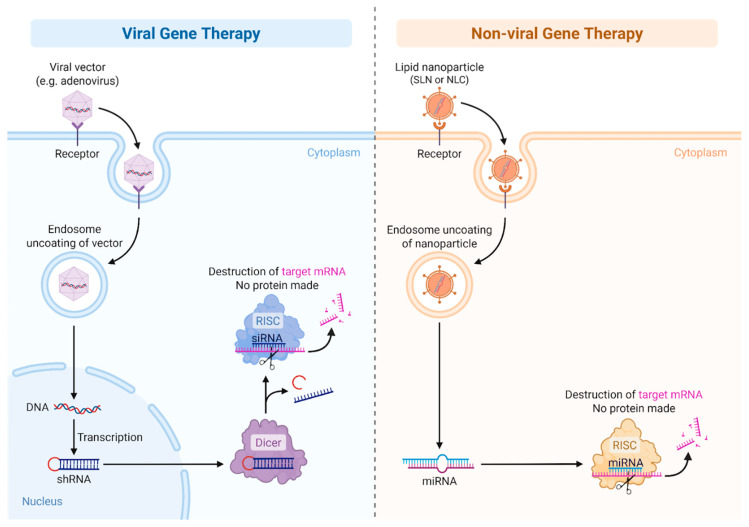
Viral vs. non-Viral Gene therapy.

Gene addition using viral vectors represents the most clinically advanced strategy in gene therapy for inherited hematologic disorders. In hemophilias A and B, adeno-associated virus (AAV) vectors are the most commonly used platforms for delivering functional copies of the F8 and F9 genes to hepatocytes. These vectors are non-integrating, replication-defective viruses that offer long-term episomal expression with minimal risk of insertional mutagenesis [64]. An illustration of viral and non-viral gene therapy is provided in Figure 3.

In hemophilia A, AAV5-based vectors like valoctocogene roxaparvovec have shown promise in clinical trials, enabling sustained expression of FVIII and reduced annualized bleeding rates. Phase 3 data have demonstrated clinically meaningful efficacy, though long-term follow-up has revealed a decline in factor VIII expression over time, suggesting durability may vary across patients [6].

For hemophilia B, AAV-mediated delivery of hyperactive F9 transgenes—particularly the Padua variant (F9-R338L)—has demonstrated high levels of FIX activity with a single intravenous infusion. Etranacogene dezaparvovec has received conditional approval by the EMA and full FDA approval based on sustained FIX activity and bleeding control over three years [65]. These therapies eliminate the need for regular factor replacement in most recipients.

However, pre-existing anti-AAV neutralizing antibodies pose a major barrier, excluding up to 30% of potential candidates. Strategies such as plasmapheresis, IgG-cleaving enzymes (e.g., IdeS), and capsid engineering are under investigation to broaden eligibility [66].

Immunogenicity to the AAV capsid or transgene product may result in hepatotoxicity or loss of transgene expression. Corticosteroids remain the mainstay for managing postinfusion transaminitis, though newer immunosuppressive regimens are being explored. Recent trials suggest that liver enzyme elevations are more frequent in AAV-FVIII gene therapy compared to FIX vectors [67].

Overall, viral gene addition has achieved regulatory success in hemophilia, but optimization of vector design, manufacturing consistency, and long-term durability remain key goals for improving outcomes in adult populations.

### 4.2. SCD/β-Thalassemia: Lentiviral Vectors with β-Globin or Anti-Sickling Globin

Lentiviral-vector-mediated gene therapy for sickle cell disease (SCD) and β-thalassemia involves the ex vivo transduction of autologous hematopoietic stem and progenitor cells (HSPCs) with vectors encoding either β-globin or anti-sickling globin variants. Unlike AAVs, lentiviral vectors integrate into the host genome, enabling long-term expression in erythroid progeny after myeloablation and autologous transplantation.

The first commercially approved lentiviral therapy for β-thalassemia, betibeglogene autotemcel (Zynteglo), uses the BB305 vector carrying a modified HBB gene. Clinical trials have shown transfusion independence in a majority of patients with transfusion-dependent β-thalassemia, with sustained vector-derived hemoglobin expression over 5 years [68]. Similar approaches have been adapted for SCD using anti-sickling variants such as HbA^T87Q, as in the LentiGlobin BB305-based therapy.

Exa-cel (exagamglogene autotemcel), a CRISPR-edited autologous product that induces fetal hemoglobin (HbF), is also manufactured using lentiviral platforms to deliver Cas9 machinery ex vivo. While distinct from traditional gene addition, these strategies share similar transplantation protocols and immune conditioning regimens.

Challenges in lentiviral therapy include the risk of insertional mutagenesis, although no leukemias have been definitively linked to vector insertion in recent trials. Furthermore, manufacturing complexity, cost, and requirement for high-dose chemotherapy pose barriers, particularly in LMICs [69].

Lentiviral therapy in SCD has shown benefit in eliminating VOCs and hemolysis markers, significantly improving quality of life. However, longer-term follow-up is needed to assess marrow engraftment durability, vector copy number stability, and the possible emergence of clonal hematopoiesis [4].

Thus, lentiviral gene therapy offers a robust platform for hemoglobinopathies, with proven curative potential, though logistical and safety challenges must be addressed to scale access globally.

### 4.3. Genome Editing Technologies: CRISPR-Cas9, Base Editors, and Prime Editing

Genome editing technologies have revolutionized the potential for curative therapy in monogenic blood disorders. CRISPR-Cas9 remains the most widely used tool, enabling precise double-stranded DNA breaks followed by repair via non-homologous end joining (NHEJ) or homology-directed repair (HDR). In SCD and β-thalassemia, this approach has focused on reactivating fetal hemoglobin (HbF) by targeting regulatory genes like BCL11A [70].

Exagamglogene autotemcel (exa-cel), developed by Vertex and CRISPR Therapeutics, uses CRISPR-Cas9 to disrupt the erythroid-specific enhancer of BCL11A, thereby releasing repression of HBG genes and restoring HbF. Phase 1/2/3 trials have shown sustained HbF induction, transfusion independence in thalassemia, and elimination of VOCs in SCD, with FDA and EMA submissions underway [71].

Beyond CRISPR-Cas9, base editors such as adenine base editors (ABEs) and cytosine base editors (CBEs) enable single nucleotide modifications without inducing double-strand breaks. This has been used to correct SCD mutations in preclinical studies and is entering clinical trials under programs like BEAM-101. These editors may reduce genotoxicity and improve safety profiles [72].

Prime editing, a more recent advancement, allows targeted insertion or correction of longer DNA sequences using a reverse transcriptase fused to Cas9-nickase. While not yet in clinical use for hemoglobinopathies, it holds promise for precise correction of complex mutations seen in β-thalassemia and compound heterozygous states [73].

Despite these breakthroughs, risks of genome editing include off-target effects, unintended indels, chromosomal rearrangements, and activation of p53 pathways. Sensitive sequencing methods are being implemented to detect low-frequency edits and improve fidelity prior to clinical release [74].

As genome editing matures, regulatory agencies continue to scrutinize safety, and long-term registries are critical for monitoring durability, malignancy risk, and hematopoietic fitness over time.

### 4.4. Targeting BCL11A Enhancer to Restore Fetal Hemoglobin

BCL11A is a transcriptional repressor of the HBG1 and HBG2 genes that encode the γ-globin chains of fetal hemoglobin (HbF). Silencing of BCL11A expression in erythroid progenitors leads to increased HbF production, which ameliorates the clinical phenotype of SCD and β-thalassemia by reducing hemoglobin polymerization and ineffective erythropoiesis.

The erythroid-specific enhancer of BCL11A, located in intron 2, has become a central target for gene therapy. Exa-cel and similar therapies use CRISPR-Cas9 to disrupt this enhancer, resulting in selective HbF induction in red blood cells without affecting immune or neuronal BCL11A expression [71].

Alternative approaches include shRNA-mediated knockdown of BCL11A via lentiviral vectors, as in the BCL11A-HPFH strategy. This method has also shown efficacy in early-phase trials, particularly for β-thalassemia patients with high transfusion requirements [70].

The degree of HbF induction correlates strongly with clinical outcomes, and threshold levels of >20% HbF have been associated with transfusion independence and elimination of VOCs. Biomarkers such as F-cell percentage and gamma-globin mRNA are being evaluated to personalize treatment intensity and predict durability [75].

Challenges remain in ensuring consistent editing rates across patient populations and stem cell harvests. Age, disease burden, and prior transfusion exposure may impact mobilization and editing efficiency. Thus, patient selection and timing are critical components of BCL11A-targeted therapy [76].

Ongoing trials are evaluating combination strategies with erythropoiesis stimulators or anti-inflammatory agents to enhance outcomes and reduce the intensity of conditioning regimens required for engraftment.

### 4.5. Risks: Off-Target Effects, Durability, Immunogenicity

One of the central concerns in gene and genome editing therapies is the risk of off-target effects. With CRISPR-Cas9, off-target cleavage may result in mutagenesis, chromosomal translocations, or oncogene activation. Advances in guide RNA design, high-fidelity Cas9 variants, and next-generation sequencing methods have improved specificity, but off-target risk cannot be fully eliminated [77].

Durability of expression is another major challenge, particularly for AAV-mediated therapies. While early trials showed high transgene expression, subsequent follow-ups revealed gradual decline in FVIII or FIX levels, potentially due to epigenetic silencing, loss of episomal vectors, or immune clearance [78]. Strategies to improve durability include codon optimization, stronger promoters, and immune modulation postinfusion.

Immunogenicity remains a significant limitation. AAV capsids elicit T-cell and humoral responses that can compromise transgene expression. Pre-existing neutralizing antibodies to AAV are common in adults and may require patient prescreening. Immunosuppression regimens using corticosteroids or sirolimus are being explored to improve vector tolerance [67].

Insertional mutagenesis is less of a concern for AAV vectors, which remain episomal, but lentiviral vectors do integrate into the host genome. Although no leukemia cases have been linked to current lentiviral platforms, clonal expansion and vector-related toxicity remain theoretical risks that require long-term follow-up [79].

Finally, manufacturing consistency, vector batch variability, and vector-associated toxicities—including hepatotoxicity and marrow suppression—are key safety endpoints monitored in late-phase trials and postapproval registries.

Balancing efficacy with safety requires comprehensive surveillance strategies, transparent data sharing, and harmonized regulatory oversight.

### 4.6. Clinical Trials and Real-World Outcomes

Over the past five years, gene therapy for hemophilia and hemoglobinopathies has progressed from clinical trials to regulatory approvals. Etranacogene dezaparvovec and valoctocogene roxaparvovec are among the first gene therapies to receive FDA/EMA approvals for hemophilias B and A, respectively. These approvals were based on trials demonstrating durable factor activity and reduction in bleeding episodes [80].

In hemoglobinopathies, exa-cel has completed pivotal phase 3 trials with regulatory submissions to the FDA and EMA. Trial results demonstrated near-complete elimination of VOCs in SCD and transfusion independence in β-thalassemia in over 85% of patients [71].

Real-world experience with gene therapies remains limited but is rapidly expanding through post-marketing registries. Emerging data have validated the safety profiles of approved therapies but also highlighted challenges such as variability in transgene expression, need for immunosuppression, and postinfusion monitoring burdens.

Limitations include high manufacturing costs, unequal global access, and the need for myeloablative conditioning. These limit scalability, especially in regions where SCD and thalassemia are most prevalent. Alternatives such as in vivo genome editing or non-viral delivery are under active investigation [72].

Furthermore, rare but serious adverse events, including delayed cytopenias and immune-mediated reactions, underscore the need for long-term monitoring. As gene therapies enter broader clinical use, real-world data will be crucial in informing guidelines, optimizing dosing, and refining patient selection criteria.

The gene therapy landscape is rapidly evolving, offering curative potential for hematologic disorders but requiring multidisciplinary infrastructure, lifelong surveillance, and ethical considerations to maximize benefit.

### 4.7. Gene Therapy for Von Willebrand Disease (VWD)

While gene therapy for hemophilia and hemoglobinopathies has made major strides, von Willebrand disease (vWD) remains largely unexplored in clinical gene therapy. vWD is genetically complex and includes both quantitative and qualitative defects of the VWF protein, complicating therapeutic design. Additionally, the large size of the VWF gene (approximately 8.4 kb coding sequence) exceeds the packaging capacity of AAV vectors (~4.7 kb), making delivery challenging.

To date, no human gene therapy trials have been conducted for vWD. However, preclinical research using murine models has demonstrated a proof of concept. A 2020 study delivered a partial-length VWF cDNA using an AAV vector, achieving partial correction of bleeding phenotype in vWD knockout mice, despite using a truncated protein [81].

The study highlighted that while truncated VWF variants can be expressed and are partially functional, complete phenotypic correction may require full-length gene delivery or alternative strategies such as dual-vector systems or lentiviral platforms. Dual AAV vector approaches, though promising, suffer from low recombination efficiency and reduced transgene expression.

Gene editing technologies are also being explored for vWD. Targeting regulatory mutations or promoting endogenous VWF expression using CRISPR-based tools may circumvent the size limitation issue. However, these approaches are in early stages of development.

Challenges include not only gene size but also VWF’s complex posttranslational processing and multimerization, which are essential for function. Any gene therapy approach must ensure proper endothelial-specific expression and intracellular processing.

Thus, while gene therapy for vWD is not yet clinically available, ongoing preclinical work supports its potential feasibility, especially with innovations in vector design and genome editing technologies.

## 5. Stem-Cell-Based Strategies

### 5.1. Allogeneic Hematopoietic Stem Cell Transplantation (HSCT)

#### 5.1.1. Applications in Hemophilias and Hemoglobinopathies

Allogeneic hematopoietic stem cell transplantation (HSCT) remains the only established curative therapy for both sickle cell disease (SCD) and transfusion-dependent β-thalassemia. It replaces the patient’s hematopoietic system with donor-derived cells that produce normal hemoglobin or coagulation factors. While not widely used for hemophilia due to the advent of effective replacement therapies, there are rare reports of factor VIII/IX normalization following HSCT performed for coexisting hematologic conditions or malignancies [82].

In contrast, HSCT is increasingly used for hemoglobinopathies, especially in pediatric patients with severe disease and access to an HLA-matched donor. In SCD, HSCT has demonstrated curative efficacy by halting vaso-occlusive events, reversing organ damage, and restoring normal erythropoiesis. In β-thalassemia, HSCT corrects ineffective erythropoiesis, eliminates transfusion dependence, and prevents iron overload progression [83].

Myeloablative conditioning with busulfan-based regimens is commonly employed, but reduced-intensity conditioning (RIC) is gaining favor in adult patients to reduce regimen-related toxicity. Treosulfan-based protocols have shown improved safety profiles with reduced hepatic and mucosal toxicity while maintaining engraftment potential [84].

Graft-versus-host disease (GVHD) remains the most serious complication, particularly in unrelated or mismatched donor settings. Acute and chronic GVHD occur in up to 30–50% of recipients depending on conditioning regimen and graft source. Prophylactic strategies such as posttransplant cyclophosphamide (PTCy) and anti-thymocyte globulin (ATG) have significantly reduced GVHD incidence, particularly in haploidentical and unrelated donor transplants [85].

Immune reconstitution following HSCT can be delayed in adults, posing risks of infection, graft failure, and secondary malignancies. Long-term outcomes are highly dependent on donor type, age at transplantation, conditioning intensity, and transplant center expertise. In thalassemia, liver iron burden and alloimmunization from prior transfusions may also influence transplant-related morbidity [86].

Despite these challenges, five-year overall survival for matched sibling donor HSCT in pediatric thalassemia and SCD exceeds 90%, and disease-free survival approaches 80–85%. In adults, outcomes are improving with careful patient selection, but late effects such as infertility and chronic GVHD remain key concerns [87].

#### 5.1.2. Matched Sibling Donor vs. Haploidentical vs. Unrelated Donors

Matched sibling donor (MSD) transplantation remains the gold standard due to lower GVHD rates, superior engraftment, and overall survival. However, only about 20–30% of patients have an available MSD, prompting the exploration of alternative donor sources, such as matched unrelated donors (MUDs) and haploidentical donors (half-matched family members) [88,89].

Outcomes in MUD HSCT have improved with high-resolution HLA typing and enhanced GVHD prophylaxis. However, GVHD risk remains slightly higher than in MSD transplants. Recent trials have shown that the use of PTCy and T-cell depletion techniques have improved the safety of MUD transplants for hemoglobinopathies [90,91].

Haploidentical HSCT has become increasingly viable due to innovations in graft engineering and GVHD prophylaxis. The use of PTCy has enabled successful engraftment with acceptable GVHD rates even in haploidentical settings. Studies report event-free survival above 70% in carefully selected SCD and β-thalassemia patients using this approach [92].

Cord blood transplantation, once considered an alternative for patients without MSDs, has seen reduced usage in hemoglobinopathies due to limited cell dose and delayed engraftment. However, in young children and select adult populations with no other donor options, cord blood remains a feasible alternative when matched appropriately [93].

Donor selection impacts not only survival and GVHD risk but also the pace of hematologic recovery and long-term immune function. GVHD remains more frequent in adults and in alternative donor settings, leading to increased reliance on supportive care, immunosuppressants, and surveillance for opportunistic infections and reactivation syndromes [94].

Future directions include the development of universal donor platforms and genetically modified allogeneic grafts that reduce HLA-mismatch-related complications. Biomarkers to personalize conditioning intensity and donor selection are also under exploration, particularly in adult patients with comorbidities or organ dysfunction.

#### 5.1.3. Autologous Gene-Modified HSC Therapy

Autologous gene-modified hematopoietic stem cell (HSC) therapy offers a transformative alternative to allogeneic HSCT by circumventing donor limitations, GVHD risk, and alloimmunization. This approach involves harvesting a patient’s own CD34+ HSCs, modifying them ex vivo—typically via lentiviral transduction or genome editing—and reinfusing them after myeloablative conditioning.

Approved platforms like betibeglogene autotemcel (for β-thalassemia) and investigational therapies like exa-cel (for both SCD and β-thalassemia) rely on either addition of a functional β-globin gene or reactivation of endogenous γ-globin via BCL11A disruption. These approaches have yielded transfusion independence or VOC elimination in the majority of treated patients [71].

A key advantage of autologous therapy is the elimination of GVHD and HLA restrictions, enabling broader applicability across ethnic and racial groups underrepresented in donor registries. Additionally, autologous therapy avoids the ethical and immunologic complexities of third-party transplantation, making it attractive for adult patients with high alloantibody burden or prior HSCT failure.

However, autologous gene therapy still requires myeloablative conditioning, most commonly with busulfan, which carries risks of infertility, cytopenias, and hepatic toxicity. Efforts to reduce conditioning intensity or develop antibody-based conditioning regimens are ongoing [95].

Another challenge is ensuring sufficient HSC harvest and transduction efficiency, especially in adult patients with chronic inflammation, prior transfusion exposure, or bone marrow fibrosis. Mobilization agents like plerixafor, along with optimized lentiviral vectors and automated manufacturing, have improved consistency and scalability [96].

Durability of engraftment and long-term hematologic reconstitution appear favorable in early follow-up data, but surveillance is critical to detect clonal dominance, vector silencing, or late marrow failure. Multiyear registries are being established to monitor for malignancy, secondary cytopenias, and genetic instability in this evolving field.

## 6. Challenges in Global Implementation

The global rollout of immune, cell, and gene therapies for hemophilias and hemoglobinopathies has been marked by stark regional disparities in regulatory approval, infrastructure, and access. Despite landmark scientific advances, the majority of patients worldwide remain unable to benefit from these potentially curative treatments. A summary of available therapies worldwide is provided in Table 1.

In Europe, the European Medicines Agency (EMA) has approved several gene and immune-based therapies. Roctavian (valoctocogene roxaparvovec), an adeno-associated virus (AAV)-mediated gene therapy for hemophilia A, received conditional EMA approval in 2022 [6]. Similarly, Hemgenix (etranacogene dezaparvovec), targeting hemophilia B, was approved in 2023 [65]. Casgevy (exa-cel), a CRISPR-edited autologous hematopoietic stem cell (HSC) therapy for sickle cell disease (SCD) and transfusion-dependent β-thalassemia (TDT), gained conditional approval in 2024 [71]. Immune therapies such as emicizumab, concizumab, and crizanlizumab are widely used across the region. Nevertheless, uptake is slowed by reimbursement hurdles, variable pricing models, and limited distribution to tertiary centers.

In Asia, progress is led by China, which approved its first domestically developed gene therapy for hemophilia B—dalnacogene macleucel (BBM-H901)—in 2023 [97,98]. While Japan and South Korea have advanced regulatory frameworks and are conducting clinical trials, no other country has approved gene or cell therapies for hemophilias or hemoglobinopathies. Access to immune therapies, particularly emicizumab, is expanding in countries like Japan, China, and India [99]. However, affordability and health system readiness remain limiting factors, especially in South and Southeast Asia.

South America faces similar challenges. No nation has approved gene therapies for these conditions. Use of immune therapies like emicizumab and crizanlizumab is growing in urban centers in Brazil, Argentina, and Colombia, often through compassionate access or private insurance channels [100]. Nonetheless, government coverage is inconsistent, and public sector adoption of high-cost therapies is rare.

Antarctica, lacking permanent inhabitants or health infrastructure, does not offer any therapies for hemophilias or hemoglobinopathies. Healthcare is restricted to emergency support for researchers and stationed personnel.

In Oceania, countries such as Australia and New Zealand have begun integrating novel therapies aligned with EMA and FDA timelines. Roctavian, Hemgenix, and Casgevy are either approved or available via special access schemes [78]. Emicizumab and crizanlizumab are routinely available in comprehensive hemophilia and sickle cell centers. However, disparities persist in rural and Indigenous populations due to geographic barriers and provider shortages.

In Africa, despite bearing over 80% of the global sickle cell disease burden, no country has approved or implemented gene therapy for SCD or thalassemia as of 2025 [101]. Access to hematopoietic stem cell transplantation is limited to a few centers in North and South Africa. Immune therapies such as emicizumab and crizanlizumab are used sparingly, often via donations or clinical trials. The situation highlights profound global inequities in curative therapy access and has raised ethical concerns regarding justice in biotherapeutic distribution [101].

These disparities underscore systemic challenges: the exorbitant cost of gene therapies (often exceeding USD 2–3 million per patient), insufficient specialized infrastructure, and fragmented regulatory pathways. While high-income countries (HICs) have benefited from early approvals and commercial rollouts, low- and middle-income countries (LMICs)—where the disease burden is greatest—continue to face marginalization. Addressing these inequities will require coordinated global efforts, including differential pricing, technology transfers, regional centers of excellence, and WHO-led equity frameworks [102].

## 7. Ethical and Societal Considerations

The deployment of gene and cell-based therapies for hemoglobinopathies and hemophilias raises complex ethical questions, particularly around informed consent, long-term surveillance, and sociocultural reception. A critical distinction exists between somatic and germline editing. While all currently approved therapies involve somatic editing—affecting only the treated individual—public discourse continues to conflate these approaches, fueling ethical concerns over heritability and intergenerational impact. Germline editing remains internationally restricted due to its ethical implications and lack of long-term safety data [103]. In contrast, somatic editing, such as CRISPR-based targeting of BCL11A in hematopoietic stem cells, is increasingly considered ethically permissible when conducted within robust regulatory frameworks and clinical oversight [71].

Informed consent in these advanced therapies must address both immediate risks and unknowns associated with long-term genomic alterations. Particularly in pediatric and adolescent populations, there are concerns about proxy consent and decisional capacity, especially when interventions carry potential lifelong biological and psychosocial implications. Consent processes must be iterative and revisited over time as new data emerge from ongoing follow-up [104].

Long-term follow-up is an ethical and regulatory imperative. Current FDA and EMA guidelines mandate decades-long surveillance post gene therapy to detect late-onset adverse events, such as insertional mutagenesis, clonal hematopoiesis, or secondary malignancy. Global registries, such as those coordinated by the World Federation of Hemophilia (WFH) and European Hematology Association (EHA), are critical for harmonizing data collection and identifying population-level safety trends [105]. However, many LMICs lack infrastructure to participate in such registries, reinforcing disparities in posttherapy monitoring.

Community perceptions of gene and cell therapies vary widely and are influenced by historical injustices, health literacy, and cultural framing of genetic interventions. In regions like sub-Saharan Africa and South Asia—where SCD and thalassemia are prevalent—concerns about experimentation, trust in biomedical institutions, and religious interpretations often shape patient receptivity. Furthermore, there is potential for stigma arising from “genetic correction” narratives, which may unintentionally pathologize disability or ethnic identity [106,107].

Ethical practice requires proactive community engagement, ensuring local stakeholders codefine research priorities, delivery models, and narrative framing. Involving patient advocates, faith leaders, and ethicists in the early stages of therapeutic deployment has been shown to enhance acceptance and adherence [108]. Moreover, equitable access remains a moral imperative: global implementation must ensure that life-saving technologies do not exacerbate health inequities between the Global North and South.

## 8. Future Directions

The next frontier of gene and cell therapy lies in multiplex editing—simultaneously modifying multiple genomic loci to achieve more robust, durable phenotypic corrections. For example, editing both BCL11A and HBG promoters may synergistically enhance fetal hemoglobin production in sickle cell disease. Tools like base editors and prime editors offer precise, low-risk multiplexing potential and are entering preclinical development for hematologic disorders [72].

Another exciting area is the creation of universal donor cell lines. By engineering allogeneic hematopoietic stem cells with immune evasion properties—such as HLA class I/II deletion or CD47 overexpression—it may be possible to create off-the-shelf gene-edited cell products for broad clinical use without the need for matched donors. Early-phase studies using gene-modified iPSC-derived progenitors demonstrate feasibility, though clinical application remains years away [109].

Synthetic biology is being leveraged to reprogram hematopoiesis and immune tolerance. For instance, synthetic promoters can be tuned to conditionally express therapeutic transgenes in response to inflammatory or erythroid-specific cues. Additionally, circuits are being developed to enable feedback-controlled expression of coagulation factors or globins, potentially improving safety and therapeutic responsiveness [110].

Integration of AI-driven modeling into gene therapy is enabling more accurate prediction of treatment responses, toxicity, and long-term outcomes. By combining genomics, transcriptomics, and clinical data, machine learning models can stratify patients for optimal gene therapy candidacy or predict vector behavior, immune responses, and off-target effects with increasing precision [111]. This approach is especially relevant in diseases with variable expressivity, like SCD or hemophilia A.

To fully realize these future strategies, sustained investment in computational biology, biomaterials, translational infrastructure, and global regulatory harmonization is essential. These tools must be deployed equitably, ensuring that innovation does not amplify existing disparities but instead becomes a lever for global therapeutic justice.

## 9. Market Withdrawal and Systemic Barriers to Access

The recent withdrawal of crizanlizumab and Zynteglo from the European market warrants brief but important reflection. Crizanlizumab, a P-selectin inhibitor approved for the reduction of vaso-occlusive crises in sickle cell disease, was withdrawn by Novartis following disappointing results from the STAND trial (NCT03814746), which failed to demonstrate a significant clinical benefit over placebo. Consequently, the European Medicines Agency (EMA) recommended revoking its conditional marketing authorization in early 2024 due to an unfavorable risk–benefit profile [47]. In contrast, the gene therapy Zynteglo (betibeglogene autotemcel) for transfusion-dependent β-thalassemia (TDT) was withdrawn from the European market in 2021 not due to clinical failure but because of economic and policy barriers. Despite receiving EMA approval, bluebird bio cited insurmountable pricing and reimbursement challenges as the primary reason for exiting Europe. These cases highlight the fragility of market access for novel therapies in Europe, where economic viability and reimbursement hurdles can derail even promising therapeutic advances.

More broadly, these developments reinforce the importance of addressing the cost of disease and the value of curative therapies, especially for lifelong, resource-intensive conditions such as β-thalassemia and sickle cell disease. In the United States, the lifetime cost of conventional transfusion and chelation therapy for TDT has been estimated at over USD 5–6 million per patient, while SCD-related hospitalizations cost the healthcare system more than USD 1.5 billion annually. In contrast, high-cost one-time curative therapies—such as gene therapy or allogeneic stem cell transplantation—may be cost-effective in the long term. For example, economic models suggest that Zynteglo, despite its ~USD 1.8 million upfront price tag, could be cost-effective under standard willingness-to-pay thresholds if long-term transfusion independence is achieved [112].

Yet, access to such therapies remains largely inequitable, particularly in regions with the highest prevalence of hemoglobinopathies. A major structural gap is the absence of robust national disease registries, which hampers surveillance, outcome tracking, healthcare planning, and cost-effectiveness analysis. A recent global survey found that only a limited number of countries, such as Iran and Cyprus, maintain functional hemoglobinopathy registries, while high-burden nations including India, Nigeria, and the Democratic Republic of Congo lack such systems entirely [113,114]. Strengthening these registries is not merely a technical exercise but a policy imperative—critical for evaluating long-term therapy outcomes, guiding research priorities, and enabling health systems to absorb the cost of curative treatments. As emphasized by international organizations such as the Thalassaemia International Federation, the integration of national registry data into healthcare infrastructure is essential to achieving equitable and sustainable access to curative therapies [115].

## 10. Conclusions

In recent years, curative therapies for hemophilias and hemoglobinopathies have progressed from scientific aspiration to clinical reality. Gene addition using adeno-associated virus (AAV) vectors, genome editing with CRISPR-Cas9, and autologous hematopoietic stem cell (HSC) therapies have demonstrated durable clinical benefits in patients with hemophilias A and B, sickle cell disease (SCD), and transfusion-dependent β-thalassemia. Immune-based therapeutics, including bispecific antibodies like emicizumab, siRNA agents such as fitusiran, and anti-adhesion biologics like crizanlizumab, have further expanded the therapeutic arsenal, offering both disease-modifying and supportive care solutions. Several of these interventions have received regulatory approval in high-income regions, marking a paradigm shift from palliative management to potential cure.

Despite these advances, significant challenges remain. Scientific and clinical questions surrounding durability, long-term safety, off-target effects, and immune responses persist. Moreover, logistical hurdles—including vector manufacturing, patient selection, infrastructure demands, and long-term follow-up—must be addressed to optimize patient outcomes. Ethical considerations around informed consent, particularly in pediatric and resource-limited contexts, and the integration of community perspectives will be crucial as the field advances toward widespread adoption.

Looking ahead, priority areas for research include multiplex gene editing, synthetic biology applications, and immune-evasive universal donor platforms. Funding must be directed not only at scientific innovation but also at scaling manufacturing, building transplant and gene therapy centers, and training providers in under-resourced settings. International collaborations—among academic institutions, regulatory agencies, industry partners, and global health bodies—will be essential to facilitate equitable trials, harmonize guidelines, and foster knowledge exchange.

Finally, achieving global health equity in the implementation of curative therapies is a moral imperative. As novel therapeutics become standard in high-income countries, deliberate action is required to prevent the widening of health disparities. Stakeholders must commit to differential pricing, regional manufacturing hubs, and inclusive regulatory frameworks. With a coordinated global effort, the promise of curative therapy can be extended to all patients—regardless of geography, income, or background—transforming the lives of those with hemophilias and hemoglobinopathies worldwide.

## Figures and Tables

**Table 1:** Global Access to Advanced Therapies for Hemophilias and Hemoglobinopathies (2025).

Region	Approved Gene Therapies	Immune Therapies Available	Key Implementation Challenges
Europe	✔ Roctavian (Hem A), Hemgenix (Hem B), Casgevy (SCD/β-thal), Zynteglo (β-thal)	✔ Emicizumab, concizumab, crizanlizumab	High cost, national reimbursement delays, infrastructure limitations
Asia	✔ BBM-H901 (Hem B—China only)	✔ Emicizumab in Japan, China, Korea; limited crizanlizumab use	Uneven regulatory progress, cost barriers, limited transplant centers
South America	✖ No approved gene therapies	✔ Emicizumab in Brazil, Argentina; limited crizanlizumab	Lack of reimbursement, delayed regulatory access, trial-dependent access
Antarctica	✖ None	✖ None	No healthcare infrastructure
Oceania	✔ Roctavian, Hemgenix, Casgevy (special access)	✔ Emicizumab, crizanlizumab	Geographic disparity, rural access limitations
Africa	✖ None	✔ Limited emicizumab, crizanlizumab via trials/donation	High disease burden, no gene therapy access, weak transplant capacity
